# Redox Imbalance in Cystic Fibrosis: The Multifaceted Role of Oxidative Stress

**DOI:** 10.3390/ph18060784

**Published:** 2025-05-24

**Authors:** Ilaria Artusi, Michela Rubin, Giorgio Cozza

**Affiliations:** 1 Department of Molecular Medicine (DMM), University of Padua, 35131 Padua, Italy; michela.rubin@studenti.unipd.it; 2 Department of Pharmaceuticals and Pharmacological Sciences (DSF), University of Padua, 35131 Padua, Italy; 3 Biostructures and Biosystems National Institute (INBB), 00165 Rome, Italy

**Keywords:** CFTR, lipid peroxidation, oxidative stress, inflammation, reactive oxygen species, radical scavengers, cystic fibrosis

## Abstract

Cystic fibrosis (CF), a severe genetic disorder stemming from mutations in the Cystic Fibrosis Transmembrane Conductance Regulator (*CFTR*) gene, is characterized by a complex interplay of chronic inflammation and heightened oxidative stress, resulting in substantial patient morbidity. The diverse array of CFTR mutations, categorized into seven distinct classes based on their functional impact on the CFTR protein, presents a significant obstacle to effective therapeutic intervention. While CFTR modulator therapies offer clinical benefits, their applicability is restricted to specific mutation classes, leaving a considerable portion of the CF patient population with unmet therapeutic needs. This review provides a critical analysis of the intricate role of oxidative stress in CF, meticulously examining its origins, mechanistic pathways and downstream pathological consequences, with particular emphasis on lipid peroxidation (LPO). It elucidates the nuanced connection between LPO and inflammatory processes driven by cellular stressors such as endoplasmic reticulum dysfunction, mitochondrial impairment and persistent bacterial infections. Furthermore, it evaluates the current landscape of therapeutic proposals targeting oxidative stress, including antioxidant interventions, and explores the potential of microRNAs (miRNAs) as novel targets. This review aims to synthesize existing research to provide a comprehensive understanding of oxidative stress involvement in CF pathogenesis while critically appraising the advantages and limitations of current antioxidant therapeutic strategies.

## 1. Introduction

Cystic Fibrosis (CF) is an autosomal recessive genetic disorder caused by mutations in the Cystic Fibrosis Transmembrane Conductance Regulator (*CFTR*) gene [1,2,3]. This gene encodes a protein that functions as a chloride and bicarbonate channel, crucial for the regulation of fluid and electrolyte transport across epithelial cell membranes. Mutations in the *CFTR* gene lead to the production of a dysfunctional or absent CFTR protein, resulting in thick, sticky mucus accumulation in various organs, mainly the lungs, pancreas and intestine [4,5,6,7,8,9]. Over 2000 mutations have been identified [10], commonly categorized into seven classes based on their impact on CFTR function, affecting protein production, processing, regulation, conductance, synthesis or turnover [11]. The most frequent mutation is represented by ΔF508. Current therapies for CF aim to manage symptoms and slow disease progression. These include airway clearance, antibiotics for infections, pancreatic enzyme replacement and CFTR modulators that target specific mutations to improve CFTR function, such as ivacaftor (VX-770), lumacaftor (VX-809), tezacaftor (VX-661) and elexacaftor (VX-445) [12]. However, their effectiveness is mutation-specific, antibiotic resistance can develop, and lung transplantation may be needed in advanced cases. Notably, Trikafta, a combination therapy including elexacaftor, tezacaftor and ivacaftor, has shown significant benefits but carries a risk of hepatotoxicity, requiring careful monitoring of liver function [13]. In December 2024, a next-generation triple combination of CFTR modulators, Alyftrek, received approval from the FDA. This therapy offers the convenience of once-daily oral administration and extends coverage to an additional 31 rare mutations [14,15,16]. Alyftrek maintains tezacaftor while incorporating a novel corrector, vanzacaftor (VX-121), and deutivacaftor (VX-561), which is the deuterated form of the potentiator ivacaftor [14,15,16]. Despite improvements in life quality and expectancy with modulators, they do not fully resolve the disease’s underlying issues.

A critical aspect of CF pathophysiology is the presence of a chronic inflammatory state accompanied by significant oxidative stress [5,17,18,19]. This imbalance arises from an overproduction of reactive oxygen species (ROS) and a compromised antioxidant defense system, contributing to cellular damage and disease progression. A particularly relevant consequence of this oxidative stress is lipid peroxidation (LPO) [20], a free radical-mediated process that affects membrane lipids. In CF, LPO can be exacerbated by factors such as endoplasmic reticulum (ER) stress [21], mitochondrial dysfunction [22] and persistent bacterial infections in the airways [23]. The interplay between LPO and inflammation is complex and likely involves a self-perpetuating cycle where LPO products further promote inflammation and the inflammatory environment enhances oxidative stress and LPO [24]. Given the substantial role of oxidative damage, including LPO, in CF pathogenesis, therapeutic strategies aimed at restoring redox balance and mitigating LPO are of considerable interest.

This review aims to exhaustively examine the manifold aspects of oxidative stress in CF, including its sources, mechanisms and consequences. Furthermore, it will explore the therapeutic strategies targeting oxidative stress in CF that have been studied so far, with a particular focus on antioxidant therapies and the potential of microRNAs (miRNAs).

## 2. Oxidative Stress in Cystic Fibrosis

Cellular redox homeostasis defines a set of finely regulated processes that maintain an equilibrium between reducing and oxidizing reactions under physiological settings. In the presence of certain physical (i.e., UV, ionizing radiations), chemical (pollutants, heavy metals, drugs) or biological (i.e., infective agents) stimuli, a release of ROS could be observed into the cells [25,26,27]. In this context, the term oxidative stress refers to a condition characterized by an imbalance between the production of ROS and the body’s ability to neutralize them [27].

ROS are unstable and extremely reactive molecules including free radicals, such as superoxide anions (O_2_^•−^) and hydroxyl radicals (HO^•^), together with non-radical species reacting as precursors of free radicals, like singlet oxygen (^1^O_2_) and hydrogen peroxide (H_2_O_2_). They play a pivotal role in mediating oxidative stress, with their effects largely dependent on their concentration and half-life. At low, physiologically controlled concentrations, such as those generated as a consequence of basal cellular metabolism, ROS act as signaling molecules stimulating adaptive cellular response and promoting homeostatic maintenance [28]. This event, termed “cellular eustress”, unfolds through the modulation of diverse cellular processes, including the antioxidant defense mechanisms, cell proliferation, differentiation, inflammatory responses and apoptosis [29]. Conversely, “cellular distress” ensues when ROS are generated in excessive quantities, leading to cellular damage via the oxidation of lipids, proteins and DNA, thereby disrupting cellular homeostasis [18].

Consequently, the presence of a redox imbalance within cells emerges as a significant contributor to the pathogenesis of numerous diseases, prominently including diabetes, cancer, neurodegenerative disorders and cardiovascular pathologies [24].

**Figure 1:**
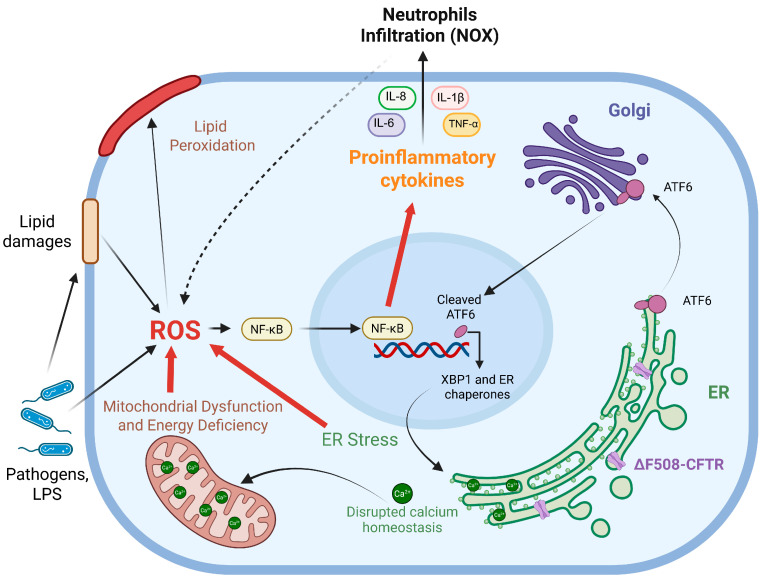
Schematic representation of pro-oxidative events initiated by a defective CFTR channel retained within the ER for subsequent degradation. As a consequence of ΔF508-CFTR impaired trafficking, oxidative imbalance was observed in CF leading to an overproduction of ROS. Primary sources of oxidative stress were: ER stress caused by mutated CFTR accumulation, persistent activation of unfolded protein response (UPR), alteration of the protein kinase RNA-like ER Kinase (PERK), the inositol-requiring enzyme 1 α (IRE1α) and the activating transcription factor 6 (ATF6) pathways, increased internal Ca^2+^ storage and the X-box binding protein 1 (XBP1)-dependent ER expansion; mitochondrial dysfunction characterized by dysregulation in Ca^2+^ signaling and anomalies in ATP production which trigger the generation of O_2_^•−^ and H_2_O_2_; and bacterial infections that promote inflammation and affect lipid homeostasis and contribute to ROS production. Oxidative imbalance is also closely related to inflammation and, in CF, a basal activation of the nuclear factor kappa-light-chain-enhancer of activated B cells (NF-κB) signaling, high levels of pro-inflammatory mediators and infiltration of neutrophils, responsible for ROS production via NOXs activity, were described. Furthermore, these oxidative events not only target protein and DNA but also damage lipid bilayers, triggering lipid peroxidation, which contributes to the self-perpetuation of these processes. Image was drawn using BioRender (https://www.biorender.com/).

Notably, an oxidative imbalance is detected also in CF, and it is thought to be strictly related to CFTR dysfunction participating in the establishment of a chronic inflammatory state, impairment of autophagy and altered lipid metabolism (Figure 1). However, this oxidative burden is further exacerbated by a combination of factors, including the accumulation of viscous mucus, persistent pathogen colonization, therapeutic antibiotic regimens and the consequential activation of host immune responses [30]. Moreover, studies have demonstrated that CFTR-deficient murine models and human lung epithelial cells exhibit diminished glutathione (GSH) levels, coupled with increased oxidative stress also in the absence of apparent pulmonary infections [31,32]. Furthermore, a study involving new-born CF pigs, which manifest lung pathology prior to measurable bacterial colonization, supports the notion of an infection-independent oxidative stress component [33]. These findings suggest that CFTR dysfunction itself instigates a fundamental redox imbalance, setting the stage for subsequent inflammatory and infectious complications. Thus, while secondary factors such as infection and inflammation undoubtedly amplify oxidative stress in CF, a primary, CFTR-driven oxidative insult appears to be a crucial initiating event.

## 3. The Connection Between Inflammation and Oxidative Stress

Inflammation and oxidative stress represent two overriding, yet intricately linked, pathological drivers in the development of lung disease in CF.

Inflammation, a fundamental immunological response to injury or infection, is designed to limit tissue damage and contain pathogen invasion. However, in CF, this defensive mechanism becomes chronically overstimulated, evolving into a significant contributor to the pathogenic state of this disease [34].

As denoted for redox imbalance, inflammatory processes manifest early in life in CF patients, even in the absence of detectable bacterial and viral infections [19]. This observation underscores the intrinsic inflammatory state established by the accumulation of thick and viscous mucus within the airways, a direct consequence of CFTR mutation and its functional impairment. This mucus traps germs and other foreign particles, thereby eliciting a sustained immune response. The subsequent activation of innate immunity recruits inflammatory cells that release proteases and other mediators, perpetuating a cycle of airway deterioration and increased susceptibility to infection.

At the molecular level, defective CFTR and the resultant proteinopathy caused by ER accumulation of misfolded proteins contribute to the basal over-expression and activation of the nuclear factor kappa-light-chain-enhancer of activated B cells (NF-κB) signaling pathway in epithelial cells. This event drives the transcription of several pro-inflammatory molecules [21,30]. Bronchial epithelial cells operate as a physical barrier and produce a variety of inflammatory agents, including cytokines, eicosanoids, enzymes and adhesion molecules. Indeed, CF lungs exhibit excessive secretion of interleukin (IL)-8, together with high levels of TNF-α, IL-1β, IL-6, IL-17A, IL-33, GM-CSF and G-CSF, accompanied by a substantial influx of neutrophils and macrophages [5,35]. The heightened production of pro-inflammatory cytokines may be attributed, in part, to leukocyte adhesion deficiency IV (LAD-IV), a defect in integrin activation observed in CF monocytes. This deficiency, coupled with the overwhelming neutrophil infiltration in CF lungs, impairs inflammation resolution and pathogen clearance [36]. Furthermore, the abundant presence of neutrophils in the bronchial fluids triggers NADPH oxidase (NOX), leading to a remarkable increase in ROS production. While bronchial ciliated and alveolar type II epithelial cells can generate ROS through DUOX1 and DUOX2, the two epithelial isoforms of NADPH oxidase, neutrophils have been identified as the major source of ROS in the airway surface liquid (ASL) of young children with CF [17,37].

The tight interplay between inflammation and oxidative stress raises a fundamental question: which precedes the other? This poses a significant challenge in dissecting the precise sequence of events in CF pathogenesis. It remains difficult to determine whether inflammation drives ROS production or if ROS, generated through various mechanisms, initiate and perpetuate inflammatory cascades. This conundrum is not unique to CF and underscores the complexity of studying chronic inflammatory diseases. As highlighted by Forman and Zhang [38], the bidirectional relationship between inflammation and oxidative stress necessitates a holistic approach to understand disease progression (Figure 1).

## 4. Sources of Oxidative Stress in CF

### 4.1. ER Stress

The ΔF508-CFTR mutation, the most prevalent CFTR mutation, results in a misfolded protein that is retained within the ER, triggering the unfolded protein response (UPR), a cellular mechanism designed to restore ER homeostasis. However, in CF, the chronic activation of the UPR leads to sustained ER stress, thereby promoting cellular dysfunction and disease progression. The UPR signaling pathways primarily involve three sensors, the protein kinase RNA-like ER Kinase (PERK), the inositol-requiring enzyme 1 α (IRE1α) and the activating transcription factor 6 (ATF6), which are activated in response to ER stress and initiate a cascade of events aimed at reducing protein misfolding and restoring ER function (Figure 1) [39]. IRE1α splices the X-box binding protein 1 (XBP1) mRNA, resulting in the active transcription factor XBP1s. XBP1s then translocates to the nucleus to upregulate genes encoding ER chaperones, folding enzymes and ER Associated Degradation (ERAD) components, enhancing protein folding and degradation to restore ER homeostasis. Furthermore, XBP1 promotes ER expansion, increasing its capacity to manage protein folding demands [40]. In CF, these pathways are often dysregulated, leading to chronic inflammation and apoptosis [41]. On the other hand, ER stress contributes to the inflammatory phenotype observed in CF, as the UPR can activate inflammatory signaling pathways such as NF-κB, responsible for the synthesis of pro-inflammatory cytokines, thus exacerbating lung injury (Figure 1) [42]. Moreover, ER stress is a significant contributor to ROS overproduction [43]. The persistent activation of UPR pathways, especially through IRE1α signaling, can lead to increased mitochondrial ROS generation. This massive ROS production further amplifies oxidative stress within CF cells, worsening cellular damage and disease progression. Furthermore, given that the ER serves as a major calcium storage organelle, CFTR mutations and ER stress disrupt calcium (Ca^2+^) homeostasis with consequent implications for mitochondrial function and cellular signaling (Figure 1, see next section for more details) [44].

Consistent with these findings, it has been demonstrated that overexpression of ΔF508-CFTR in a Calu3 cell model induces ER stress via UPR. This UPR activation, in turn, leads to a marked decrease in CFTR mRNA levels, establishing a detrimental feedback loop that further compromises CFTR activity [45]. Moreover, the involvement of the UPR transducer, ATF6, was reported in A549 cells transfected with ΔF508-CFTR compared to wt-CFTR. Specifically, a reduced expression of ATF6 seems to be correlated with an increased CFTR localization at the PM, thus suggesting ATF6 as a possible therapeutic target, as its modulation could potentially enhance CFTR trafficking and function [46].

### 4.2. Mitochondrial Dysfunction

Mitochondrial dysfunction emerges as a pivotal contributor to the pathogenesis of CF, exacerbating both oxidative stress and inflammatory responses. Decreased mitochondrial GSH concentration coupled with increased mitochondrial DNA oxidation and reduced aconitase activity, an enzyme sensitive to oxidative inactivation, was reported in both in CFTR KO mice and human lung epithelial cells harboring mutated CFTR [32]. These findings underscore the direct involvement of mitochondria in the genesis of oxidative stress within CF. The dysregulation of mitochondrial function further amplifies ROS production, thereby triggering the activation of the inflammasome and pro-inflammatory transcription factor NF-κB. This cascade culminates in the augmented levels of pro-inflammatory cytokines, including IL-1β, TNF-α, IL-6 and IL-17A [47,48]. In CF, these events determine the so called “mito-inflammation” [49].

Intriguingly, a heightened oxygen consumption rate was observed in ΔF508-CFTR cells, which may be attributed to increased O_2_^•−^ and H_2_O_2_ production [50]. This observation is consistent with reports of impaired mitochondrial respiratory chain complex I and IV activities, as well as dysregulation of the ADP/ATP exchanger, further contributing to mitochondrial ROS generation and increased lipid peroxidation (Figure 1) [22].

Furthermore, mitophagy, the selective degradation of damaged mitochondria, is demonstrably defective in fibrotic diseases, including CF. Given the critical role of mitophagy in balancing oxidative stress, inflammatory responses, cellular dynamics regulation and energy metabolism, its impairment significantly contributes to fibrotic progression [51,52].

The effects of mutated CFTR extend beyond the pulmonary system, as evidenced by studies demonstrating mitochondrial dysfunction and lipid homeostasis disruption at the intestinal level. For example, these perturbations in a *CFTR*-knockout Caco2 cell line were observed, indicating systemic implications of CFTR mutations [53].

Finally, it is crucial to recognize the intricate interplay between ER stress, mitochondrial dysfunction and lipid homeostasis (as discussed in Section 6) in the establishment of an oxidative and inflammatory status in CF. As highlighted by Kumar and Maity, ER stress-sensor proteins (IRE1α, PERK, ATF6) not only initiate the UPR, as discussed previously, but also directly modulate mitochondrial dynamics and function. For instance, IRE1α can interact with mitochondrial proteins, influencing their morphology and respiratory capacity. Furthermore, the ER-mitochondria associated membranes (MAMs) serve as critical platforms for calcium and lipid transfer, and their disruption in CF contributes to both ER and mitochondrial dysfunction [54]. In particular, in CF, aberrant Ca^2+^ signaling, due to ER stress, disrupts mitochondrial buffering capacity, leading to abnormal and increased Ca^2+^ uptake within these organelles (Figure 1). This, in turn, impacts mitochondrial ATP production, a process heavily reliant on Ca^2+^-dependent enzymes within the tricarboxylic acid (TCA) cycle. Consequently, CF cells often exhibit impaired mitochondrial ATP synthesis, resulting in cellular energy deficits. Furthermore, where baseline oxidative stress is already elevated due to CFTR loss-of-function, dysregulated Ca^2+^ signaling exacerbates ROS production, increasing oxidative damage. Additionally, Ca^2+^ signaling finely controls mitochondrial dynamics, including fusion and fission. Disruptions in Ca^2+^ homeostasis in CF lung disease compromise these processes, leading to mitochondrial fragmentation. Moreover, abnormal Ca^2+^ signaling increases cellular susceptibility to apoptosis, contributing to tissue damage [55].

### 4.3. Bacterial Infections

In CF lung, *Pseudomonas aeruginosa* stands as a primary and persistent pathogen, and its presence triggers and sustains the inflammatory state. This exacerbated inflammation, in turn, contributes to ER stress, specifically the UPR, driven by the accumulation of inflammatory mediators within the organelle (Figure 1). The manifestation of ER stress is characterized by increased internal Ca^2+^ storage and XBP1-dependent ER expansion (see previous sections). Notably, *P. aeruginosa* and *Staphylococcus aureus* have been shown to induce IL-8 secretion in airway epithelial cells through a Ca^2+^ mobilization-dependent mechanism, thereby influencing mucin secretion [21,56]. Complementarily to these findings, Kunzelmann and colleagues demonstrated that *P. aeruginosa* infection induces an increase in ROS, causing lipid peroxidation and cell death in airway epithelial cells, independent of CFTR genotype. Furthermore, they observed that CF cells, specifically those expressing the ΔF508-CFTR mutation, fail to activate fluid secretion in response to *P. aeruginosa* infection, a phenomenon attributed to dysregulated Ca^2+^ signaling and subsequent impairment of Ca^2+^-dependent adenylate cyclase activation [55]. Adding to this understanding of infection-mediated pathogenesis, recent research has highlighted how host cell metabolic alterations could promote bacterial persistence. Specifically, studies indicated that the combined effect of PTEN (phosphatase and tensin homolog deleted on chromosome 10) and CFTR dysfunction stimulates mitochondrial activity, leading to the excessive release of succinate and ROS [57]. This altered metabolic environment in the CF airway, characterized by elevated succinate levels, favors the colonization of *P. aeruginosa*, which can preferentially metabolize this substrate [57]. Moreover, this metabolic shift induces an anti-inflammatory host response dominated by IRG1 (immune-responsive gene 1) and itaconate production. Despite this response, the recruitment of myeloid cells is inefficient in clearing the infection, leading to an accumulation of phagocytes. Further complicating the host–pathogen dynamics, the survival strategies employed by *P. aeruginosa* within immune cells contribute to the persistent inflammatory burden. A study elucidated the temporally dynamic role of superoxide dismutase B (SODB) in *P. aeruginosa* survival during macrophage phagocytosis. SODB initially participates in the generation of lethal levels of H_2_O_2_ within the phagosome. However, at later stages of infection, SODB facilitates bacterial survival, linked to the activation of autophagy in macrophages. This SODB-dependent induction of autophagy, triggered by early H_2_O_2_ production, appears to be exploited by the bacteria to promote their intracellular persistence. These findings underscore the intricate network between bacterial infection, host cell signaling, metabolic reprogramming, immune cell responses and specific bacterial enzymatic activities in the context of the settled inflammatory state observed in the CF lung [58].

## 5. Oxidative Stress Can Lead to Lipid Peroxidation

LPO occurs when cellular oxidative damage specifically affects lipid bilayers, with the resulting oxidation of membrane polyunsaturated fatty acids (PUFAs), the main players in this process [59].

**Figure 2:**
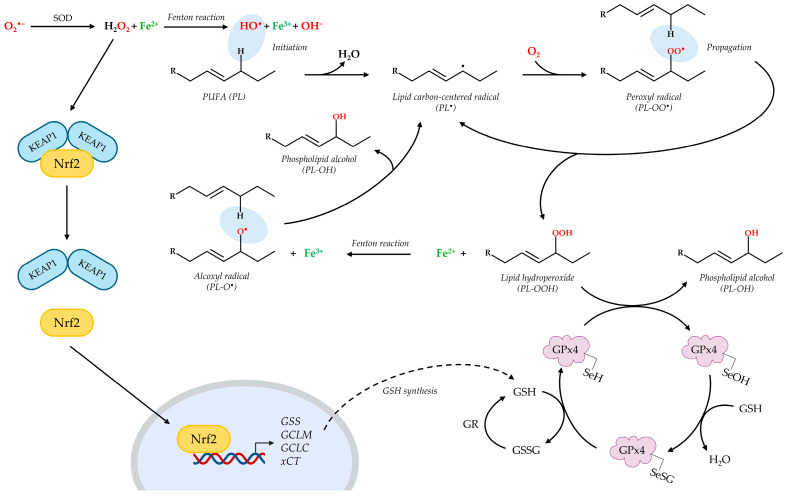
Schematic depiction of lipid peroxidation and its regulation by the Nrf2/GSH/GPx4 axis. During the Fenton-like reaction, hydrogen peroxide (H_2_O_2_) reacts with ferrous iron (Fe^2+^) to obtain the hydroxyl radical (HO^•^). The initiation of LPO involves the interaction of HO^•^ with a polyunsaturated fatty acid phospholipid (PL) that generates a carbon-centered radical (PL^•^). The rapid reaction with molecular oxygen leads to the intermediate formation of a peroxyl radical (PL-OO^•^), which reacts with another PL to produce a new PL^•^, thus propagating the process, and a lipid hydroperoxide (PL-OOH). The Fenton-like reaction then produces the alkoxyl radical (PL-O^•^) from PL-OOH; PL-O^•^ acts as a novel initiator of the radical chain reaction. The principal defensive mechanism against LPO is represented by the GPx4-GSH axis, in which the GPx4 enzyme reduces PL-OOH to the non-toxic alcoholic form (PL-OH). Then, selenium in its active site is regenerated in a two-step reaction by GSH. GSH synthesis is regulated by the transcription factor Nrf2. Increased levels of ROS induce a conformational change in KEAP1 that releases Nrf2, which can translocate into the nucleus and recognize the ARE sequences. This interaction induces the transcription of antioxidant genes such as *GSS, GCLM* and *GCLC* (homonymous subunits of the GCL), involved in GSH synthesis, and *xCT*. Image was generated using PowerPoint (Microsoft 365 suite), with the exception of icons and chemical structures, which were imported from BioRender and BIOVIA Draw 24.1 (https://www.3ds.com/products/biovia/draw).

The initiation of LPO involves the interaction of a highly reactive free radical, the hydroxyl radical (HO^•^), with membrane phospholipids (PLs). This HO^•^ can be generated through the Fenton reaction, wherein hydrogen peroxide (H_2_O_2_) reacts with ferrous iron (Fe^2+^). Furthermore, O_2_^•^⁻, particularly those originating from mitochondrial respiration, can contribute to LPO. Superoxide can either be directly involved in radical reactions or dismutate to form H_2_O_2_, thereby fueling the Fenton reaction and subsequent HO^•^ generation (Figure 2). The abstraction of a hydrogen atom from a phospholipid by these initiating radicals generates a carbon-centered radical (PL^•^). This radical rapidly reacts with molecular oxygen, leading to the intermediate formation of a peroxyl radical (PL-OO^•^). The process then propagates via the reaction of PL-OO^•^ with another PL to produce a new PL^•^ and a lipid hydroperoxide (PL-OOH). The presence of Fe^2+^ then triggers the formation of the alkoxyl radical (PL-O^•^) from a pre-existing PL-OOH. The extremely reactive PL-O^•^ is competent as a new initiator of the oxidative events leading to a significant amplification of the LPO process (Figure 2) [60].

Uncontrolled lipid radical chain reactions produce a multitude of oxidized lipid species via termination reactions (the coupling of two lipid radicals), events that contribute to cellular damage and ultimately culminate in ferroptosis, an iron-dependent form of regulated cell death distinguished morphologically, biochemically, and genetically from apoptosis, necroptosis, and pyroptosis [61,62,63]. Unlike apoptosis, which is characterized by caspase activation and cellular shrinkage, or necroptosis, which involves receptor-interacting serine/threonine kinase (RIPK) activation and membrane rupture, ferroptosis is uniquely defined by the iron-dependent accumulation of lipid peroxides to lethal levels, ultimately leading to non-lytic cell death [64]. Furthermore, ferroptosis exhibits distinct biochemical features, such as the inactivation of the selenoprotein glutathione peroxidase 4 (GPx4) and the absence of canonical apoptotic or necroptotic signaling cascades [62]. Intracellular accumulation and bioavailability of iron are critical determinants in the progression of LPO and its potential evolution into ferroptosis. After cellular uptake of extracellular transferrin-bond iron through transferrin receptor (TFR), ferric iron (Fe^3+^) is then internalized in endosomes and reduced to its ferrous form (Fe^2+^) by the iron oxide reductase STEAP3. Divalent metal transporter 1 (DMT1) facilitates the transport of Fe^2+^ into the cytoplasm where, if not stored as Fe^3+^ by ferritin, it maintains its redox-active state, participating in the cellular labile iron pool (LIP) [65].

Interestingly, an impairment in iron homeostasis was observed in CF patients since high levels of iron were noticed in bronchoalveolar lavage fluid (BALF), and increased expression of ferritin and DMT1 was reported in lung tissues [66].

In addition to the non-enzymatic, iron-mediated, lipid autoxidation, PUFA oxidation can also be catalyzed by lipoxygenases (LOXs) or cytochrome P450 oxidoreductase (POR) activity, as recently and extensively discussed in a comprehensive review [67].

Against ferroptosis, two major enzymes involved in the detoxification of hydroperoxides from phospholipids and cholesterol have been identified: GPx4 performs an essential task in catalyzing the reduction of PL-OOH, normally produced in trace amounts during aerobic metabolism, into corresponding phospholipid alcohols (PL-OH), by using GSH as reducing agent [68]. The indispensable nature of GPx4 in suppressing ferroptosis is underscored by the observation that its inactivation or depletion is sufficient to trigger this form of cell death. Additionally, the ferroptosis suppressor protein 1 (FSP1) regenerates non-mitochondrial ubiquinone (Coenzyme Q10, CoQ10), which traps and neutralizes lipid peroxyl radicals [69].

A key role in the cellular antioxidant response is played by GSH, mainly as cofactor of GPx4. The mechanism initiates with the active site of GPx4 containing a selenocysteine residue in its reduced form (GPx4-SeH). Reduced GPx4 reacts with a PL-OOH, resulting in the reduction of the hydroperoxide to a PL-OH and the oxidation of the selenocysteine to a selenenic acid (GPx4-SeOH). To restore the enzyme’s catalytic activity, two molecules of GSH are required. The first GSH reacts with the selenenic acid to obtain a selenenyl sulfide intermediate (GPx4-SeSG) and a molecule of water. Subsequently, a second GSH reduces the selenenyl sulfide intermediate, regenerating the active reduced form of GPx4 (GPx4-SeH) and forming glutathione disulfide (GSSG) [70,71]. This cyclical process ensures the continuous detoxification of lipid hydroperoxides, provided that sufficient levels of GSH are available to maintain GPx4 in its active state and to manage the resulting GSSG (Figure 2) [72,73]. Being an ABC superfamily member, CFTR shares some structural similarities with multidrug resistant proteins (MRPs), known exporters of GSH [18]. So, it is not surprising that CFTR activity contributes, among other factors, to the maintenance of a high GSH concentration in the airway lumen (300–800 μM), where it exerts its antioxidant activity against inflammatory and infectious stimuli. However, this concentration is markedly reduced to approximately 10% in CF patients [74]. The intracellular storage of GSH is further regulated by the uptake and synthesis of its constituent amino acids. Precisely, the cystine/glutamate antiporter Xc^−^, located at the plasma membrane, mediates the import of cystine, which is subsequently reduced to cysteine, a rate-limiting precursor for GSH synthesis. Concurrently, the de novo synthesis of GSH is tightly controlled by the nuclear factor (erythroid-derived 2)-like 2 (Nrf2), the main regulator of the antioxidant response element (ARE) pathway (Figure 2) [75]. Generally associated with the inhibitory protein Kelch-like ECH-associated protein 1 (KEAP1), Nrf2 is released and translocates to the nucleus in response to oxidative stress-induced modifications of KEAP1 [76]. Within the nucleus, Nrf2 binds to the ARE promoter sequence, thereby stimulating the transcription of genes involved in the antioxidant response, drug detoxification, NADPH regeneration and metabolism regulation [76]. Among others, Nrf2 directly influences the expression of genes encoding enzymes crucial for GSH synthesis, including the catalytic (GCLC) and modifier (GCLM) subunits of glutamate cysteine ligase (GCL), together with glutathione synthetase (GSS) [77,78]. Furthermore, Nrf2 also regulates the expression of the system Xc^−^ light chain (xCT), thus modulating cystine uptake and GSH synthesis (Figure 2) [79].

GSH deficiency is strictly connected to CFTR loss-of-function, contributing significantly to the compromised antioxidant defenses observed in CF patients. Specifically, a marked increase in the GSSG relative to reduced GSH is observed in the ASL of CF patients. This shift in the GSH/GSSG redox balance reflects a diminished capacity to neutralize ROS and maintain cellular redox homeostasis. Moreover, considering that CFTR has been established to facilitate the transport of GSH across cellular membranes, the defective activity of CFTR in CF directly impairs the efflux of GSH into the ASL, leading to its depletion and exacerbating oxidative stress [5,30].

On the other hand, Chen and others demonstrated that in CF epithelia, the expression and activity of Nrf2 are decreased by ~70% when compared to non-CF cells. They concluded that a decrease in Nrf2 in CF cells leads to an increase in H_2_O_2_ levels, which in turn promotes the overproduction of pro-inflammatory cytokines [80].

In addition to this, a reduced activity of glutathione reductase (GR), involved in the recycling of GSSG into GSH, was observed in ΔF508-CFBE cells, together with increased NOX activity and DUOX2 protein levels compared to cells expressing wt-CFTR. Both enzymes consume NADPH as substrate, the first to decrease GSH and the second to produce anion superoxide from oxygen, thereby contributing to ROS production out of mitochondria [81].

Dysregulation of Nrf2 and antioxidant enzyme activity is responsible for the decrease in GSH synthesis and secretion to the lung lumen, corroborating the inflammatory state and pathogen proliferation. Insufficient levels of GSH that cause a constant antioxidant deficit also promote unrestrained LPO, causing widespread oxidative damage to cells and tissues and leading to the onset of ferroptosis. The connection between LPO, ferroptosis and chronic airway inflammation has recently emerged. Specifically, low levels of GPx4 and GSH have been associated with the development of chronic obstructive pulmonary disease (COPD) and the insufficient antioxidant stress response of human bronchial epithelial cells when exposed to cigarette smoke [82]. Additionally, ferroptosis has been found to significantly participate in the mechanism of bronchial asthma exacerbation through further introduction of PL-OOH by 5- and 15-lipoxygenase [83].

Focusing on CF, it was reported that *P. aeruginosa* employs its 15-lipoxygenase (pLoxA) to induce LPO and ferroptotic death in bronchial epithelial cells. This process occurs through two mechanisms: first, by oxidizing the host’s arachidonoyl-lipid into the hydroperoxyl derivatives and second, by suppressing the host’s GPx4 defense system [84]. Relative to this, CF epithelia show an intrinsic pro-inflammatory phenotype, and ferroptosis was observed in CF patients after pulmonary bacterial infections especially induced by pathogens like *P. aeruginosa* [23], suggesting a role of this recently emerged type of cell death in the progression of the pathology.

Indeed, a recent study highlighted that IB3-1, a CF epithelial cell line, demonstrates an increased susceptibility to cell death when exposed to divalent iron or erastin, a ferroptosis inducer [85].

## 6. Altered Lipid Metabolism and Lipid Imbalance in CF

Many studies underlined the relation between CFTR deficiency and altered lipid metabolism, which, in turn, is responsible for tissue remodeling, exacerbation of a pro-oxidative and inflammatory environment and impaired autophagy in CF airways [5].

In detail, a study comparing different CF models (ΔF508 mouse lungs, *CFTR* KO pigs and primary human bronchial epithelial cells) correlated oxidative stress with an increased ω-6- tο ω-3-PUFA ratio and abnormal levels of sphingolipids, especially ceramides, as long-chain to very long-chain ceramide species (LCC/VLCC) [86]. The same results were obtained from lipidomic analyses on a bronchial epithelial IB3-1 cell model [87] or in BALF samples from young children with CF [86].

Sphingolipids are bioactive lipids that, in the outer membrane, are organized in lipid rafts and contribute, together with cholesterol, to CFTR stability and activity. However, in CF, an increased amount of sphingolipids was detected at the apical membrane compared to non-CF ALI cultures, and the activity of enzymes implied in their catabolism seemed to be impaired [88,89,90]. Again, their composition drastically changed in favor of ceramides obtained from the catabolism of sphingomyelin or glucosylceramide due to bacterial activity [89].

As a member of the ABC transporters, which are involved in lipid transport and regulation of membrane integrity, a role of CFTR in the maintenance of lipid bilayer stability could be speculated. Indeed, it is known that when the expression of the ATP binding cassette transporter A1 (ABCA1) is repressed, an excess of cholesterol builds up in alveolar cells, damaging surfactant function and increasing the inflammatory response that was implicated in the pathogenesis of COPD, asthma and other lung diseases [91,92].

In addition, an augmented release of arachidonic acid (AA) from cell plasma membrane and an alteration in the levels of its precursor, linoleic acid (LA), was also reported in CF. On the other hand, a decreased amount of resolving ω-3 docosahexaenoic acids (DHA) was detected, indicating a high AA/DHA ratio in CF samples. Disturbances in annexins and ceramides might act in concert, explaining the impact on inflammation and AA release [93,94]. Alteration in AA metabolism in CF then triggers an imbalance between pro-inflammatory eicosanoids, as leukotrienes, and pro-resolving metabolites, called lipoxins, corroborating the development of chronic inflammation [34].

These changes in lipid metabolism definitely modify the lipid storage and cell membrane composition and, together with pathogen activity, stimulate the synthesis of pro-inflammatory lipid species contributing to the lung disease.

## 7. Therapeutic Approaches Against Oxidative Stress in CF

**Figure 3:**
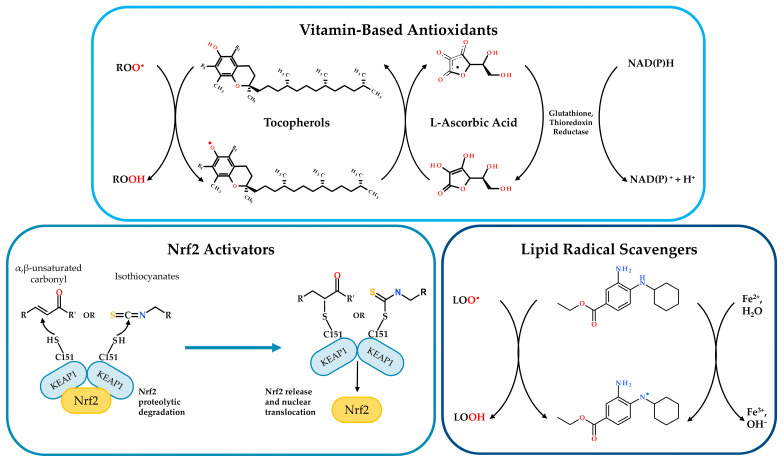
Illustration of current research approaches to counteract oxidative stress in CF and other inflammatory diseases. Vitamin-Based Antioxidants: Tocopherols (vitamin E) and L-ascorbic acid (vitamin C) directly scavenge ROS. Tocopherols neutralize peroxyl radicals (R-OO^•^) to form tocopheroxyl radicals and hydroperoxides (R-OOH). L-ascorbic acid reduces tocopheroxyl radicals, regenerating tocopherols and forming ascorbyl radicals. Glutathione reductase and thioredoxin reductase, utilizing NADPH, regenerate reduced L-ascorbic acid. Nrf2 Activators: α,β-unsaturated carbonyls and isothiocyanates modulate Nrf2 pathway. Under basal conditions, Nrf2 is bound to KEAP1, leading to its proteolytic degradation. Activators modify KEAP1, resulting in Nrf2 release, nuclear translocation and subsequent transcription of antioxidant genes. Lipid Radical Scavengers: A representative lipid radical scavenger compound (ferrostatin-1) reacts with the alkoxyl radical (L-O^•^), forming the alcohol derivative L-OH. The process involves the generation of water and the oxidation of ferrous iron (Fe^2+^) to ferric iron (Fe^3+^), as described in [60]. Image was generated using PowerPoint (Microsoft 365 suite); chemical structures were imported from BIOVIA Draw.

Given that oxidative stress is crucial in the pathogenesis of CF, significantly supporting chronic inflammation and progressive lung damage as elucidated in the previous sections, the redox imbalance correction represents a therapeutic strategy for improving CF outcomes. In the literature, diverse interventions have been proposed to address this issue, yielding varied results (Table 1). These approaches can be broadly categorized into the modulation of the Nrf2/GSH axis, the use of vitamin-based antioxidants or compounds targeting LPO and lipid imbalance and the application of specific microRNAs (Figure 3).

### 7.1. Activators of Nrf2/GSH Signaling Pathway

Considering the low levels of both Nrf2 and GSH in CF models and the crucial role of the latter in controlling lipid peroxidation, a therapeutic approach based on Nrf2 activators could prove promising. Under physiological conditions, Nrf2 is sequestered in the cytoplasm by KEAP1, which facilitates its ubiquitination and subsequent proteasomal degradation, thereby keeping Nrf2 levels low in the absence of oxidative stress. However, when cells encounter oxidative or electrophilic stress, the Nrf2/KEAP1 interaction is disrupted, allowing Nrf2 to translocate into the nucleus and induce the expression of ARE target genes, including those encoding the enzymes involved in GSH synthesis. A significant number of Nrf2 activators function as Michael acceptors, which are electrophilic compounds capable of reacting with nucleophilic residues, particularly cysteine thiols [95]. In Michael addiction reactions, two important classes of Michael acceptors are (i) α,β-unsaturated carbonyls and (ii) isothiocyanates. α,β-Unsaturated carbonyls contain a carbon–carbon double bond conjugated with a carbonyl group. This chemical feature makes the β-carbon electrophilic and susceptible to nucleophilic attack. Precisely, the cysteine thiol group attacks the electrophilic β-carbon of the α,β-unsaturated carbonyl, resulting in the formation of a covalent adduct between the cysteine residue and the carbonyl compound (Figure 3) [96]. Isothiocyanates are organosulfur compounds characterized by the -N=C=S functional group where the central carbon is electrophilic due to the cumulative electron-withdrawing effect of the nitrogen and sulfur atoms. The nucleophilic attack by the thiol group generates a dithiocarbamate adduct (Figure 3) [97].

These classes of molecules target specific cysteine residues on KEAP1, as Cys151, inducing a conformational change that compromises its ability to bind and sequester Nrf2. This modification of KEAP1 effectively stimulates the antioxidant defense system by promoting Nrf2 activation and subsequent transcription of its target genes [98,99].

Curcumin, for instance, contains two α,β-unsaturated carbonyl groups, and it has recently been demonstrated to activate Nrf2 through this mechanism [100]. Some studies have investigated the potential of curcumin as a therapeutic agent for CF, especially in addressing the ΔF508 mutation. Early research demonstrated promising results in both in vitro and in vivo models. Indeed, baby hamster kidney cells transfected with human ΔF508-CFTR exhibited improved processing of the mutant protein when exposed to curcumin. Furthermore, the same work reported that oral administration of curcumin to ΔF508 CF mice resulted in a significant correction of ΔF508-CFTR trafficking [101]. However, subsequent attempts to replicate these findings yielded inconsistent results. Notably, a follow-up investigation failed to duplicate the nasal potential-difference measurements observed in the initial study, as well as the corrective effects of curcumin in vitro [102]. A recent study suggested that, by restoring Nrf2 activity, curcumin treatment normalizes inflammation in CFTR-depleted zebrafish and prevents tissue damage by promoting collagen fiber remodeling. Similarly to curcumin, resveratrol, a polyphenolic compound containing α,β-unsaturated carbonyl groups and possessing Nrf2-activating properties [103], manifested initial promise as a potential therapeutic agent for CF. Preliminary studies reported encouraging outcomes, including an enhanced conversion of band B to band C for ΔF508-CFTR in Western blot analysis, indicating improved protein maturation and trafficking [104]. Additionally, resveratrol was observed to elicit salutary effects, such as increased airway fluid secretion and enhanced mucociliary clearance, which are crucial for maintaining proper lung function in CF patients [104,105]. However, as with curcumin, further investigations provided conflicting results. In a study utilizing primary human airway epithelial cells derived from patients homozygous for the ΔF508-CFTR mutation, researchers were unable to confirm the beneficial effects of resveratrol exposure. This discrepancy is particularly noteworthy given that known CFTR “correctors” demonstrated efficacy in the same experimental model [106]. More recently, the Nrf2 activator dimethyl fumarate (DMF), exploited for its anti-inflammatory and antioxidant effects, was observed to modulate the inflammatory response upon LPS stimulation in both CF and non-CF bronchial epithelial cells. DMF can reinstate pro-inflammatory cytokine expression and ROS levels in CF cells bearing the most common mutation ΔF508 (both CFBE and HNE cells), and it restores Trikafta™ efficacy, decreased due to the LPS effect, thus working in association with and addition to CFTR modulators [107]. It was also pointed out that CFTR modulators, specifically lumacaftor and tezacaftor, induce a significant augmentation of Nrf2 activity in individuals affected by CF [108]. Nevertheless, the currently proposed mechanism appears to be indirect, as Nrf2’s reinstated activity is a consequence of the restoration of CFTR protein to the cell membrane by the two correctors [108].

Beyond the activation of Nrf2 to increase GSH levels and decrease LPO, GSH supplementation itself has been tested in recent years as a therapy to reduce the oxidant burden in CF patients. In CF isolates, GSH was able to disrupt *P. aeruginosa* biofilms by inactivating the metabolite pyocyanin, which contributes to ROS production [109]. However, different clinical trials [110,111] highlighted that, even though well tolerated, GSH inhalation did not improve lung function (FEV improvement) or ameliorate oxidative stress and inflammation. A significant reduction in BALF PGE2 levels and higher numbers of lymphocytes were recorded instead, suggesting an effect on the modulation of the immune response [112]. The efficacy of the presented therapy had limitations linked to sputum levels of γ-glutamyltransferase, an enzyme secreted by activated phagocytes that rapidly degrades GSH.

On the other hand, GSH oral administration produced controversial outcomes: a beneficial effect on growth and inflammatory status at the gut level was observed only in pediatric patients developing a moderate disease [113] but not in children with pancreatic insufficiency [114].

Considering GSH temperature instability and rapid oxidation, its precursor N-acetylcysteine (NAC) has also been taken into account in recent times. NAC could be exploited for its antioxidant activity and, at lower doses, to modulate airway inflammation and prevent biofilm formation, in particular, that triggered by *P. aeruginosa.* Administration by inhalation was observed to provide great efficacy [115]. Other formulations of NAC, including *N*-acetylcysteine amide (NACA) or (2R,2R′)-3,3′-disulfanediyl bis(2-acetamidopropanamide) (diNACA), have been considered to enhance the drug’s bioavailability for the prevention and treatment of radiation-induced pneumonia and of pulmonary function in CF, COPD, or bronchitis. Furthermore, diNACA has been evaluated for the reduction of mucus viscosity and/or elasticity [116].

### 7.2. Vitamin-Based Antioxidants

One of the main consequence of CFTR loss of function at the intestinal level is malabsorption of lipids and fat-soluble vitamins, especially in CF patients suffering from pancreatic insufficiency. As a consequence, supplementation of antioxidants and vitamins has been investigated in recent years, and their effectiveness in ameliorating CF patients’ quality of life has also been explored [117].

On the one hand, different studies demonstrated that oral administration of antioxidants [118], β-carotene [119] or antioxidant-enriched multivitamins [120] did not provide an improvement in lung function. On the other hand, an increase in antioxidant blood levels, with the exception of ascorbic acid (vitamin C), is generally described.

For instance, antioxidant-enriched multivitamins were orally supplied to pancreatic-insufficient CF patients where a reduced absorption of dietary antioxidants, including carotenoids such as β-carotene, tocopherols (vitamin E), CoQ10 and selenium was usually observed [120]. At week 4, a systemic increase in antioxidant concentration was reported, associated with a modest reduction in circulating amounts of calprotectin and myeloperoxidase (MPO), indicators of inflammation. Conversely, MPO levels in the sputum did not change. No improvement in lung function and growth was even observed; the treated group had a lower risk of first pulmonary exacerbation correlated with a reduced need for antibiotics compared to the control group [120].

Ascorbic acid displayed the ability to inhibit bacterial quorum sensing, thus affecting chemotaxis and biofilm formation (Figure 3). In vitro analyses highlighted that ascorbic acid notably impaired *Bacillus subtilis*, *Escherichia coli* or *P. aeruginosa* biofilm formation [121], and destruction of *Mycobacterium abscessus* biofilms was also documented [122]. One study investigated plasma concentrations of vitamin E and A after one-year treatment with lumafactor/ivafactor. Beyond improvements in lung function and pulmonary inflammatory state, plasmatic levels of vitamin A significantly increased, while slight decreases in vitamin E and the vitamin E/cholesterol ratio were reported [123].

### 7.3. Molecules Addressing LPO or Lipid Imbalance

Given the heightened LPO observed in CF models, alongside the striking imbalance in fatty acid and ceramide metabolism associated with chronic oxidative stress under basal conditions in both CF mouse lung tissue and patients, a novel therapeutic approach could potentially involve molecules capable of targeting and inhibiting LPO. Indeed, deferoxamine, an iron chelator with the capacity to inhibit LPO, was able to partially correct the lipid profile [86]. Ferrostatin-1 [60], a well-known scavenger of lipid radicals, has been shown to block peroxidative damage and ferroptotic cell death induced by *P. aeruginosa* in mice lacking CFTR expression (Figure 3) [23]. Although ferrostatin-1 is not currently considered a clinical drug candidate, its application in research settings suggests that its antioxidant mechanism, which directly targets specific lipid radicals, may represent a promising avenue for future investigation into the inflammation and infection paradigms in CF.

Noted to increase the synthesis and reduce the catabolism of lipids in CF, fenretinide, a synthetic retinoid, showed the capacity to address the fatty acid imbalance observed in CF. While its mechanism of action has not yet been directly correlated with the inhibition of LPO, it is noteworthy that this compound, by modulating the expression of mucins, particularly MUC5AC and MUC5B, which are crucial in CF pathology, in a manner similar to clinically used CFTR modulators, exhibited beneficial effects on lipid homeostasis and redox balance [124]. On the other hand, sphingolipid synthesis inhibitor myriocin (Myr) significantly decreased the previously described dyslipidemia, with a positive effect on the modulation of the immune and inflammatory response [125]. Addressing the topic of sphingolipid modulation in CF, recent investigations have noted that the elexacaftor/tezacaftor/ivacaftor (ETI) combination therapy perturbs the de novo sphingolipid biosynthesis pathway in primary human bronchial epithelial cells [126]. A particularly striking observation is a doubling of dihydrosphingolipid levels [126]. Mechanistically, subsequent studies have revealed that tezacaftor acts as an inhibitor of the sphingolipid delta-4 desaturase enzyme (DEGS), resulting in a consequential accumulation of dihydroceramides relative to ceramides [127]. This identified off-target effect necessitates thorough evaluation to ascertain potential long-term deleterious consequences.

**Table 1:** Overview of various antioxidant therapeutic approaches in cystic fibrosis, summarizing therapeutic categories, mechanisms of action, findings, and future prospects.

Therapeutic Approach	Mechanism of Action	Model System(s) Tested In	Effects of Treatment	Prospects for Improvement
***Activators of Nrf2/GSH signaling pathway***				
**General Nrf2 Activators (Michael Acceptors) [95,96,97,98,99]**	Interaction with KEAP1 cysteine residues (Michael reaction), Nrf2 translocation to nucleus and induction of ARE target genes (incl. GSH synthesis enzymes).	General cellular mechanism description.	Stimulation of the antioxidant defense system.	Nrf2 activators are considered potentially promising. No effect on CFTR function in single treatment.
**Curcumin [100,101,102]**	Nrf2 activation (Michael reaction against KEAP1). Investigated for addressing ΔF508 mutation.	In vitro: Baby hamster kidney cells (ΔF508-CFTR transfected). In vivo: ΔF508 CF mice. CFTR-depleted zebrafish.	*Initial studies*: Improved ΔF508-CFTR processing (in vitro); corrected ΔF508-CFTR trafficking (mice). *Later studies*: failed to replicate initial findings. *Recent studies*: Restored Nrf2 activity (zebrafish), normalized inflammation.	Inconsistent results highlight challenges; potential benefits in specific contexts like inflammation and tissue damage.
**Resveratrol [103,104,105,106]**	Nrf2 activation (Michael reaction against KEAP1).	In vitro: Primary sinonasal epithelial cells from humans, mice, and pigs: CFBE (wt, ΔF508-CFTR) and HEK293T-CFTR expressing either wild type or F508del CFTR In vivo: wt and ΔF508-CFTR mice	*Initial promise*: Improved ΔF508-CFTR maturation/trafficking; increased airway fluid secretion and mucociliary clearance. *Conflicting results*: Beneficial effects not confirmed in primary human airway cells from patients.	Conflicting results cast doubt on its consistent efficacy as a therapeutic agent for CF.
**Dimethyl fumarate (DMF) [107]**	Nrf2 activation (Michael reaction against KEAP1); anti-inflammatory and antioxidant effects. Modulation inflammatory response to LPS.	CF (ΔF508: CFBE, HNE cells) and non-CF bronchial epithelial cells.	Reinstated pro-inflammatory cytokine expression and ROS levels in ΔF508 CF cells; restored Trikafta™ efficacy reduced by LPS. Association with CFTR modulators.	Promising as an adjunctive therapy with CFTR modulators, particularly in managing inflammation.
**GSH Supplementation [109,110,111,112,113,114]**	Direct increment of GSH levels; reduction of oxidant burden; inactivation of *P. aeruginosa* metabolite pyocyanin.	*P. aeruginosa* biofilms (CF isolates); Clinical trials (human inhalation); Pediatric patients (oral adm.); Children with pancreatic insufficiency (oral adm.).	*Biofilms*: Disrupted *P. aeruginosa* biofilms. *Inhalation*: Well tolerated, but no improvement in lung function, oxidative stress, or inflammation; reduced BALF PGE2, higher lymphocytes (immune modulation). *Oral*: Controversial; some benefit in pediatric patients with moderate disease, not in those with pancreatic insufficiency.	Inhalation limited by degradation and lack of lung function improvement. Oral route shows highly specific and limited benefits. Overall efficacy remains a challenge.
**N-acetylcysteine (NAC) [115]**	GSH precursor; antioxidant; modulation of airway inflammation; prevention of biofilm formation (*P. aeruginosa*).	CF patients, clinical investigation/use.	Inhalation observed to provide great efficacy.	Considered promising, especially via inhalation.
**NACA** **(*N*-acetylcysteine amide) and diNACA [116]**	Formulations to enhance NAC bioavailability. diNACA also for reducing mucus viscosity/elasticity.	Implied for CF, COPD, bronchitis, radiation-induced pneumonia.	Being evaluated.	Potential to enhance NAC’s therapeutic benefits by improving bioavailability and mucolytic effects (diNACA).
***Vitamin-based antioxidants***				
**Antioxidants, β-carotene, Antioxidant-enriched multivitamins [119,120]**	As supplement to counteract malabsorption of fat-soluble vitamins and antioxidants.	CF patients (pancreatic-insufficient for multivitamins).	Generally, no lung function improvement (oral adm.). Increased blood antioxidant levels. Multivitamins: modest reduction in some inflammation markers; no lung improvement; lower risk of first pulmonary exacerbation and reduced antibiotic need.	No impact on lung function, but potential improvement of systemic antioxidant status and reduced exacerbation frequency.
**Ascorbic acid (vitamin C) [121]**	Inhibits bacterial quorum sensing, affecting chemotaxis and biofilm formation.	In vitro: *B. subtilis*, *E. coli*, *P. aeruginosa*, *M. abscessus* biofilms.	Impaired biofilm formation and promoted destruction of existing biofilms.	Interesting in vitro antibiofilm activity; clinical relevance and efficacy in CF patients require further investigation.
**Vitamin A and E status [123]**	Observation of vitamin levels during CFTR modulator treatment.	CF Patients under lumacaftor/ivacaftor treatments.	Plasma vitamin A significantly increased; Vitamin E and Vit E/cholesterol ratio slightly decreased (alongside improved lung function from modulators).	Need to monitor and adjust vitamins supplementation during CFTR modulator therapies due to altered vitamins levels.
***Molecules addressing LPO or lipid imbalance***				
**Deferoxamine [86]**	Iron chelator; inhibition of Lipid Peroxidation (LPO).	CF and non-CF primary cells from human epithelium; primary cell from CF mice (Cftr^tm1eur^ F508del) and pigs (CFTR-KO)	Partially correction of lipid profile.	Potential use if iron-mediated LPO significantly contributes to CF pathology; further research needed.
**Ferrostatin-1 [23,60]**	Scavenger of lipid radicals	Mice lacking CFTR expression (with *P. aeruginosa* infection).	Blocked peroxidative damage and ferroptotic cell death induced by *P. aeruginosa*	Not a current clinical drug candidate, but its mechanism is promising against CF inflammation and infection.
**Fenretinide [124]**	Modulation of fatty acid imbalance and MUC5AC and MUC5B expression.	CF patients; Mice lacking CFTR expression (with *P. aeruginosa* infection).	Exhibited beneficial effects on lipid homeostasis and redox balance.	Potential positive effects on lipid and redox equilibrium; it warrants further investigation.
**Myriocin [125]**	Sphingolipid synthesis inhibitor.	CF cell models (IB3-1 cells, primary cells ΔF508/W1282X)	Significantly decreased dyslipidemia; positive effect on modulating immunity and inflammatory response.	Promising for addressing CF-associated dyslipidemia and its impact on inflammation and immunity.

### 7.4. MicroRNAs as Potential Targets to Regulate Oxidative Stress

MicroRNAs (miRNAs) are small, non-coding, endogenous RNAs of about 20 nucleotides involved in the modulation of many biological processes, such as cell proliferation and metabolism, through the synthesis and suppression of several target genes [128]. They modulate the translation and stability of their target mRNAs and, in turn, protein expression by binding to a complementary sequence located in the 3′-untranslated region (3′-UTR) [129]. Recent evidence has positioned miRNAs as predictive biomarkers for human pathologies, and they are also increasingly recognized as strategic therapeutic targets [130].

Modulation of miRNAs levels can be achieved with miRNA mimics, synthetic double-stranded small RNAs that amplify or restore their activity, or with antagonists, called antimiRNAs, which reduce the function of these biomolecules if overexpressed. They could be represented by small-molecule inhibitors, miRNA sponge, containing multiple complementary sequences of the miRNA of interest, and again ASOs, antisense oligonucleotides that directly bind miRNAs, avoiding their interaction with the target [131].

Although several miRNAs responsible for the regulation of channel activity have been identified [132], the design of miRNA-based therapies specifically directed against CFTR synthesis is complicated by the phenotypic variability of CF patients bearing the same genotype, by the variety of clinical manifestations and by the fact that CFTR expression is tissue-specific and time-dependent [133]. As a consequence, attention has shifted to different targets involved in several other aspects of the pathology.

**Figure 4:**
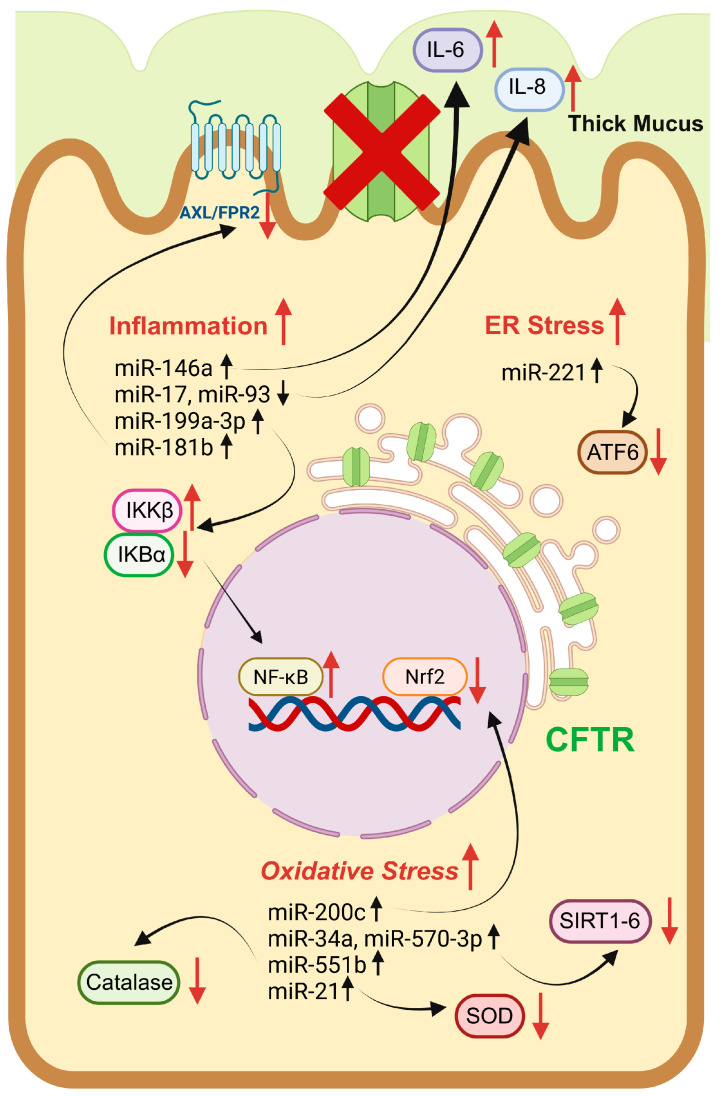
Pictorial representation of key miRNAs implicated in the regulation of ER stress, mitochondrial dysfunction, and inflammation in a CF model. miRNAs can influence cellular targets with diverse functions, including enzymatic proteins (e.g., catalase, SOD, sirtuins, and IKK), transcriptional factors (e.g., ATF6, Nrf2, and NFkb), and inflammatory modulators (e.g., IL-6 and IL-8). Red upward arrows indicate induction of cellular processes or protein expression instead red downward arrows suppression. Black upward and downward arrows denote increased and decreased miRNA level, respectively. The red cross symbol over the CFTR channel means its functional absence at the plasma membrane. Image was drawn using BioRender (https://www.biorender.com/).

Actually, several altered miRNAs have been identified in CF cell lines that critically contribute to the inflammatory process [134] (Figure 4, Table 2).

For example, pro-inflammatory cytokines IL-6 and IL-8 are overproduced, the first from CF macrophages upon LPS stimulation in response to high levels of miR-146a [135], and the second as a consequence of miR-17 and miR-93 deregulation in bronchial epithelial cells infected by *P. aeruginosa* [136,137] and, again, of miR-199a-3p, which also increases IKKβ expression that, in turn, hyperactivates the NF-κB pathway in CF airway epithelial cells [133].

Focusing on lipid mediators, the ALX/FPR2-dependent pathway, activated by lipoxin LXA_4_ and resolvin RvD_1_ and implicated in inflammation resolution, is altered by the increased synthesis of miR-181b in CF lung cells and macrophages (Table 2) [138].

Among regulators of cellular oxidative stress response, non-coding RNAs (microRNAs, long non-coding RNAs, circular RNAs) recently have been recognized as important positive and negative modulators [139]. Indeed, it was observed that ROS can influence the expression of specific microRNAs, and, at the same time, these biomolecules can act as redox-sensitive nuclear factors, thus stimulating gene expression of specific enzymes and proteins involved in the antioxidant response [140] (Table 2).

For example, in pulmonary diseases such as COPD, oxidative stress, and, in particular, augmented H_2_O_2_ concentration via phosphoinositide-3-kinase (PI3K) signaling, increases miR-34a levels, with the consequent reduction in both mRNA and the amounts of sirtuin-1 and -6 (SIRT1/-6) deacetylases. This effect causes the activation of ageing responses and induction of cell senescence [141]; in the same pathology, miR-570-3p is also highly expressed under marked oxidative stress and p38 MAPK activity, thus causing, as for miR-34a, downregulation of anti-aging enzyme SIRT1 expression (Table 2) [142].

Many antioxidant enzymes are also regulated by miRNAs: catalase expression is decreased by miR-551b in a cell model of lung cancer [143], while miR-21 is responsible for downregulation of intracellular and extracellular SOD3 levels and indirect TNFα-mediated reduced production of mitochondrial SOD2 in human bronchial cells (Table 2) [144].

Glutathione peroxidase expression and activity seem to be strictly related to the upregulation of specific miRNAs that can, depending on the disease and cellular model studied, both decrease GPxs expression or increase their activity [140]. Moreover, the cellular concentration of their cofactor GSH is indirectly regulated by these RNA-based biomolecules by influencing Nrf2 levels and activation. One, miR-200c, oversees the oxidative stress response by inhibiting Nrf2 expression in a lung cancer cellular model [145].

Moreover, ER stress also is observed to be affected by miRNA regulation: in bronchial epithelial cells, miR-221 is particularly responsible for decreasing ATF6 levels and the control of airway inflammation (Table 2) [146].

The use of miRNAs as therapeutic targets constitutes a compelling new approach in the context of emerging RNA-based therapies, and several clinical trials focused on this technology, for treatment of diverse pathologies, are in progress [147]. However, some limitations have emerged, including miRNA toxicity, interaction with off-targets, design complexity and, as a consequence, unwanted side effects. More deeply, their application in CF encounters substantial obstacles related to effective delivery to pulmonary target cells that is significantly hindered by the tenacious CF mucus; inherent challenges in cellular internalization; and the necessity to ensure target specificity and mitigate pleiotropic off-target effects stemming from the capacity of miRNAs to modulate numerous gene targets. The innate instability of miRNA molecules, making them susceptible to rapid enzymatic degradation and conferring a short in vivo half-life, often requires chemical modifications that themselves may introduce further complications or elicit adverse immunogenic reactions [148]. Therefore, proper delivery strategies should be developed to overcome these issues. Moreover, the intricate and multifactorial pathophysiology of CF, characterized by widespread dysregulation of miRNA expression, poses considerable challenges for the rational selection of therapeutic candidates.

**Table 2:** Key miRNAs implicated in the regulation of the indicated biological processes, examined within the context of lung disease models; increase (↑) or decrease (↓) miRNA level is highlighted.

Pathway	miRNAs	Outcome	Reference
Inflammation	↑ miR-146a	IL-6 overproduction	[135]
	↓ miR-17 and mi-R93	IL-8 overproduction	[136,137]
	↑ miR-199a-3p	IKKβ increased expression and NF-κB hyperactivation	[133]
	↑ miR-181b	Alteration of ALX/FPR2-dependent pathway	[138]
Oxidative stress	↑ miR-34a	SIRT1/-6 deacetylases decreased expression	[141]
	↑ miR-570-3p	SIRT1 deacetylase decreased expression	[142]
	↑ miR-551b	Downregulation of catalase expression	[143]
	↑ miR-21	Downregulation of SOD3 and TNFα	[144]
	↑ miR-200c	Inhibition of Nrf2 expression	[145]
ER stress	↑ miR-221	Downregulation of ATF6 levels	[146]

To conclude, despite the difficulties encountered in the development of miRNA therapeutics, their use in the clinical setting still arouses interest, and synergistic treatments with miRNAs and CFTR modulators for CF patients that do not sufficiently respond to the approved therapies have been suggested [149].

## 8. Conclusions

Oxidative stress represents a crucial aspect in CF disease, especially in relation to inflammation and bacterial infection. A phenomenon of particular interest is LPO, a radical reaction involving membrane lipids that, in CF, can be correlated with ER stress, mitochondrial dysfunction or bacterial infection. The timing between LPO and inflammation is not easily clarified; certainly, there is a strong correlation between the two events, but it appears to be more of a circular effect than a linear one, with each stimulating the other and vice versa. Therefore, it becomes particularly interesting to consider therapeutic approaches that target redox imbalance and LPO. Given the dependence of GPx4, the enzyme responsible for removing membrane hydroperoxides, on glutathione levels and the observed low levels of both GSH and Nrf2 in CF, stimulating the Nrf2/GSH axis could represent a promising antioxidant strategy. However, while modulation of the Nrf2/GSH signaling pathway presented initial promise with compounds like curcumin and resveratrol, subsequent investigations often failed to consistently replicate these early optimistic findings, highlighting challenges in translational efficacy. DMF has shown some capacity to modulate inflammation and improve CFTR modulators’ beneficial effects under inflammatory conditions. Furthermore, CFTR modulators themselves can indirectly enhance Nrf2 activity, suggesting potential for combinatorial approaches. On the other hand, direct GSH supplementation has faced limitations due to rapid degradation and inconsistent clinical benefits in lung function, though its precursor, N-acetylcysteine (NAC), appears more consistently promising for its antioxidant and mucolytic properties, particularly in inhaled formulations.

Similarly, even well-tolerated and with no side effects, vitamin-based antioxidant molecules substantially failed in CFTR repair. Despite this, antioxidants have demonstrated a predominant effect on rebalancing the inflammatory state, and they seemed to reduce disease progression and pulmonary exacerbation mainly if administered at an early stage of the disease. Nevertheless, the translation of in vitro experimental findings to in vivo models is frequently marked by substantial outcome discrepancies. Such divergences are largely ascribed to the complex physiological environment of living organisms, encompassing murine models and human subjects. Within these systems, therapeutic agents encounter notable challenges, including effectively crossing biological membranes, achieving optimal systemic distribution and resisting metabolic inactivation, factors considerably less pertinent or altogether absent in controlled in vitro settings. Indeed, from clinical trials that have been carried out so far, results regarding the effectiveness of antioxidants were not unequivocally obtained, and a correct dosage and timing of administration need to be determined. It must be noted that all these studies lack in-depth mechanistic approaches, failing to assess, for example, the potential decrease in LPO during treatments, although plausible given the known mechanisms of action of these molecules. As a consequence, while the rationale for antioxidant intervention in CF is strong, the efficacy of such agents is highly variable, and initial preclinical promise often does not translate directly to consistent clinical improvement. Currently, antioxidant molecules do not appear to directly augment CFTR channel function; rather, their potential utility is recognized in their capacity to mitigate the pro-oxidative and pro-inflammatory cascades inherent to CF pathophysiology. Nevertheless, it is imperative that larger and focused clinical studies be conducted to definitively define the beneficial effects of antioxidants in CF.

On the other hand, compounds that directly interfere with membrane radicals have been scarcely studied in CF, with the exception of ferrostatin-1, which has shown optimistic potential against *P. aeruginosa* infections. Even ferrostatin-1 cannot represent a drug option for its chemical features, compounds exhibiting the same mechanism of action against lipid radicals could represent hopeful alternatives. In this context, other molecules capable of correcting dysregulated lipid homeostasis, like fenretinide and myriocin, have already proven effective in reducing inflammation.

An innovative strategy could be targeting miRNAs, particularly those associated with a decrease in primary antioxidant defense and an increase in inflammation. To date, only a few clinical trials based on miRNA therapeutics have been performed since this approach entails specificity, stability and toxicity issues, and deeper investigations, such as on the proper design and delivery of miRNAs molecules, are needed.

In conclusion, to date, the remodeling of oxidative damage at the level of cell membranes and, more generally, the rebalancing of the altered oxidative state in CF have been specifically investigated with respect to inflammation or infectious events, while the aspect of channel function remains rather elusive. Despite this, considering the tight connection between defective CFTR and oxidative stress, it should be noted that the combined approach of antioxidant molecules with various formulations of correctors and potentiators has shown positive therapeutic effects in CF patients and could represent an approach to be further explored in the near future.

## Data Availability

No new data were created or analyzed in this study. Data sharing is not applicable to this article.

## Abbreviations

The following abbreviations are used in this manuscript:

^1^O_2_	Singlet oxygen
AA	Arachidonic acid
ARE	Antioxidant response element
ASL	Airway surface liquid
ATF6	Activating transcription factor 6
BALF	Bronchoalveolar lavage fluid
CF	Cystic fibrosis
CFBE	Cystic fibrosis bronchial epithelial
CFTR	Cystic Fibrosis Transmembrane Conductance Regulator
COPD	Chronic obstructive pulmonary disease
CoQ10	Coenzyme Q10
DHA	Docosahexaenoic acids
ER	Endoplasmic reticulum
ETI	Elexacaftor/tezacaftor/ivacaftor
Fe^2+^	Ferrous iron
Fe^3+^	Ferric iron
GCL	Glutamate-cysteine ligase
GCLC	Glutamate-cysteine ligase catalytic subunit
GCLM	Glutamate-cysteine ligase modifier subunit
GPx4	Glutathione peroxidase 4
GR	Glutathione reductase
GSH	Glutathione
GSS	Glutathione synthetase
GSSG	Glutathione disulfide
H_2_O_2_	Hydrogen peroxide
HNE	Human nasal epithelial
IL	Interleukin
IRE1α	Inositol-requiring enzyme 1 α
KEAP1	Kelch-like ECH-associated protein 1
LOXs	Lipoxygenases
LPO	Lipid peroxidation
miRNAs	MicroRNAs
MRPs	Multidrug resistant proteins
NAC	*N*-acetylcysteine
NF-κB	Nuclear factor kappa-light-chain-enhancer of activated B cells
NOX	NADPH oxidase
Nrf2	Nuclear factor (erythroid-derived 2)-like 2
O_2_^•−^	Superoxide anions
HO^•^	Hydroxyl radicals
PERK	Protein kinase RNA-like ER Kinase
PL-O^•^	Alkoxyl radical
PL-OH	Phospholipid alcohols
PL-OO^•^	Peroxyl radical
PL-OOH	Lipid hydroperoxide
PLs	Phospholipids
PM	Plasma membrane
PUFAs	Polyunsaturated fatty acids
ROS	Reactive oxygen species
SOD	Superoxide dismutase
UPR	Unfolded protein response
XBP1	X-box binding protein 1
xCT	System Xc^−^ light chain
